# Onset of occupational hand eczema among healthcare workers during the SARS‐CoV‐2 pandemic: Comparing a single surgical site with a COVID‐19 intensive care unit

**DOI:** 10.1111/cod.13618

**Published:** 2020-06-16

**Authors:** Anne Guertler, Nicholas Moellhoff, Thilo L. Schenck, Christine S. Hagen, Benjamin Kendziora, Riccardo E. Giunta, Lars E. French, Markus Reinholz

**Affiliations:** ^1^ Department of Dermatology and Allergy University Hospital, LMU Munich Germany; ^2^ Division of Hand, Plastic and Aesthetic Surgery University Hospital, LMU Munich Germany

**Keywords:** COVID‐19, hand eczema, healthcare workers, irritant contact dermatitis, occupational, prevention measure, SARS‐CoV‐2

## Abstract

**Background:**

As a result of the COVID‐19 outbreak, hygiene regulations have been revised and hand sanitation has been intensified.

**Objective:**

To investigate the onset of hand eczema during the COVID‐19 pandemic in healthcare workers (HCWs) directly involved in intensive care of COVID‐19 patients and HCWs without direct contact with COVID‐19 patients. Hereby, we aim at increasing awareness about occupational hand eczema and preventive measures that can be adopted.

**Method:**

A survey was distributed amongst 114 HCWs at a single surgical centre and at a COVID‐19 intensive care unit of the university hospital Ludwig Maximilian University Munich, Germany. Participants were questioned about the daily frequency of hand hygiene prior to and during the pandemic. Participants self‐reported the onset of hand eczema and associated symptoms.

**Results:**

Our study revealed a significant increase in hand washing, disinfection, and use of hand cream across all participants (*P*‐value <.001), regardless of having direct contact with COVID‐19 patients. A high prevalence of symptoms associated with acute hand dermatitis of 90.4% was found across all HCWs, whereas hand eczema itself was underreported (14.9%).

**Conclusion:**

The increase in hand sanitation during the COVID‐19 pandemic impairs the skin of the hands across all HCWs, independent of direct intensive care of affected patients.

## 1 INTRODUCTION

“Wash your hands frequently with sufficient soap and warm water for at least 20‐30 seconds” has become an omnipresent mantra during the global COVID‐19 pandemic. The World Health Organization recommends an increase of personal hygiene in order to “flatten the curve” of infections and to prevent healthcare facilities from collapsing.^1^ In line with this, hospitals have urged healthcare workers (HCWs) to adopt increased hygiene safety precautions. Specifically, requirements include sufficient personal protective equipment (PPE) and an increase in the frequency of hand washing and disinfection.^2^ While these measures are indispensable to prevent transmission of COVID‐19, they also have negative implications.

For example, excessive sanitizing of hands using soap or alcohol‐based products leads to a disruption of the skin flora and the natural protective skin barrier.^3^, ^4^, ^5^ This accounts for a large number of occupational skin diseases (OSDs), mostly irritant and allergic contact dermatitis.^6^, ^7^, ^8^, ^9^, ^10^, ^11^ While hand eczema affects all HCWs, surgeons and surgical nursing staff are especially prone to the disease.^12^, ^13^ Obviously, healthy hands are particularly important for a surgeon and any impairment imposes significant limitations on their ability to practice.^14^, ^15^ Recently, reports have addressed cutaneous complications related to increased hand hygiene measures and prolonged time of wearing of PPE amongst HCWs treating patients with COVID‐19.^16^, ^17^, ^18^, ^19^ However, we hypothesized that an exacerbation of occupational skin injury across all disciplines might be observed, independent of having direct contact with patients that have tested positive to SARS‐CoV‐2.

The present study evaluated whether the recommendations for increased hand sanitation affected HCW’s hand washing and disinfection frequency at a single surgical centre. Additionally, we investigated whether this correlated with an onset of hand eczema and associated symptoms. Moreover, our results were compared to HCWs involved in direct intensive care of COVID‐19 patients.

## 2 MATERIALS AND METHODS

### 2.1 Design of questionnaire

Basic information regarding gender, age, occupation (physician or nurse), smoking behaviour, and clinical history of type IV hypersensitivity and atopic diathesis (asthma, allergic rhinitis, atopic dermatitis) were obtained. The present condition of the hands during the pandemic was self‐evaluated with regard to possible onset of hand eczema and associated symptoms such as erythema, itching, scaling, pain, burning, fissures, dryness, and other symptoms. Participants were questioned about the daily frequency of hand washing and hand disinfection and their responses were rated as follows: <5x =1, 5–10x =2, 10–20x =3, 20–30x=4, >30x=5, and for hand cream application: 0x=1, 1x=2, 1‐2x=3, 2‐5x=4, 5–10x=5, >10x=6, prior to, and during, the COVID‐19 pandemic.

### 2.2 Collection of data

Ethical approval was obtained by the ethics committee of the medical faculty of the Ludwig Maximilian University (LMU), Munich, Germany (Ref.‐No. 20‐330). Patient information and identification were kept confidential at all times and data analysis was performed anonymously. The survey was distributed to 114 HCWs in the first 2 weeks of April 2020. The study population included surgeons and nurses from a single surgical centre and physicians and nurses from an intensive‐care unit (ICU) for COVID‐19 patients. Therefore, HCWs were divided into two groups: a non‐COVID (Non‐C) and COVID (C) group.

### 2.3 Statistical evaluation

A professional statistician performed all data analysis using SPSS software version 26 (IBM, Armonk, New York). A *P*‐value of .05 was set as the level of significance for all statistical tests. Highly significant results were defined as *P*‐value <.01. Descriptive statistics (mean, median, standard deviation, minimum, maximum) for demographic data were calculated. Non‐parametric tests such as Mann‐Whitney *U* test, Wilcoxon test, chi‐square test, and Fisher's exact test were applied as appropriate.

## 3 RESULTS

### 3.1 Demographic data

The study population included 114 HCWs (39 physicians, 75 nurses) with a mean (±SD) age of 35.23 ± 10.78 years. The demographics of both groups (Non‐C and C) are presented in Table 1. The C group included 40 participants (21 females, 19 males; mean age 32.00 ± 6.85 years), and was significantly younger than the Non‐C group (n = 74, 49 females, 25 males; mean age 36.96 ± 12.07; *P* = .007). Both groups were comparable regarding the distribution of physicians and nurses (~1:3; *P* = .59). Smokers were distributed evenly between both groups (*P* = .33).

**TABLE 1:** Characteristics of the study population according to treatment of COVID‐19 patients

	Non‐COVID	COVID	Total	*P*‐value
Participants	n = 74	n = 40	n = 114	
Female, n (%)	49(66.2)	21(52.5)	70(61.4)	.11
Male, n (%)	25(33.8)	19(47.5)	44(38.6)	
Mean age (±SD) (years)	36.96 (±12.07)	32.00 (±6.85)	35.23 (±10.78)	.007
Range of age (y)	43	37	43	
Physicians, n (%)	24(32.4)	15(37.5)	39(34.2)	.59
Nurses, n (%)	50(67.6)	25(62.5)	75(65.8)	
Smoker, n (%)	27(36.5)	11(27.5)	38(33.3)	.33
Type IV hypersensitivity, n (%)	8(11) n = 73	2(5)	10(8.8)	.49
Asthma, n (%)	7(9.5)	3(7.5)	10(8.8)	> .99
Allergic rhinitis, n (%)	21(28.4)	8(20.5) n = 39	29(25.7) n = 113	.36
Atopic dermatitis, n (%)	8(10.8)	2(5)	10(8.8)	.30

Abbreviations: SD, standard deviation

### 3.2 Type IV hypersensitivity and atopy

Overall, HCWs reported type IV hypersensitivity, asthma, allergic rhinitis, and atopic dermatitis in 8.8%, 8.8%, 25.7% and 8.8% of all cases, respectively. The Non‐C group showed slightly higher shares of type IV hypersensitivity (11% vs 5%) and atopic diathesis (asthma: 9.5% vs 7.5%; allergic rhinitis: 28.4% vs. 20.5%; atopic dermatitis: 10.8% vs 5%), but differences were all non‐significant (*P* > .05). (Table 1).

### 3.3 Symptoms associated with hand eczema

Our data show a high prevalence of self‐reported symptoms associated with hand eczema across all HCWs. Dryness was reported most frequently (83.2%), followed by erythema (38.6%), itching (28.9%), burning (21.1%), scaling (18.4%), fissures (9.6%), and pain (4.4%. (Table 2). Comparison of the Non‐C and C group revealed no significant difference in self‐reported symptoms between the groups. Interestingly, although the majority of participants suffered from symptoms associated with hand eczema (90.4%, n = 103/114), only 14.9% of HCWs (n = 17/114) actively recognized the symptoms as an onset of the disease. (Tables 2 and 3) Of these participants, 70.6% (n = 12/17) reported the date of onset, and this revealed that only two HCWs suffered from hand eczema for more than 50 days. (Table 4).

**TABLE 2:** Self‐reported symptoms of hand eczema according to treatment of COVID‐19 patients

Symptoms	Non‐COVID n = 74 n (%)	COVID n = 40 n (%)	Total n = 114 n (%)	*P*‐value
Erythema	31(41.9)	13(32.5)	44(38.6)	.33
Itching	21(28.4)	12(30)	33(28.9)	<.001
Scaling	16(21.6)	5(12.5)	21(18.4)	.31
Pain	4(5.4)	1(2.5)	5(4.4)	.66
Burning	19(25.7)	5(12.5)	24(21.)	.10
Fissures	8(10.8)	3(7.5)	11(9.6)	.74
Dryness	62(83.8)	32(82.) n = 39	94(83.2) n = 113	.82
Other	3(4.1) n = 73	1(2.5)	4(3.5)	>.99
≥ 1 symptom	69(93.2)	34(85.0)	103(90.4)	.19

**TABLE 3:** Self‐reported onset of hand eczema according to treatment of COVID‐19 patients

Self‐reported diagnosis	Non‐COVID n = 74	COVID n = 40	Total n = 114	*P*‐value
Hand eczema, n (%)	11(14.9)	6(15)	17(14.9)	.99

**TABLE 4:** Onset of hand eczema across all healthcare workers

Presence of hand eczema (days)	n	%	Cumulative %
<10	1	8.3	8.3
11–20	3	25.0	33.3
21–30	4	33.3	66.6
30–50	2	16.7	83.3
>50	2	16.7	100
Total	12	100	

### 3.4 Hand washing and disinfection

The overall frequency of hand washing before and during the pandemic showed a highly significant increase from 5–10x per day to 10–20x per day for all HCWs (*P* < .001). (Table 5) Both groups (Non‐C and C) were comparable with regard to hand washing frequency, and showed no significant difference (*P* = .70) (Table 6).

**TABLE 5:** Median daily frequency of hand washing, disinfection, and application of hand cream across all health care workers before and during the COVID‐19 pandemic

	Before COVID‐19	During COVID‐19	*P*‐value
Hand washing	2.0 n = 113	3.0 n = 113	<.001
Hand disinfection	3.0 n = 113	4.0 n = 113	<.001
Hand cream	2.0 n = 114	3.0 n = 114	<.001

*Note*: Mean Hand washing and disinfection frequency <5x =1, 5–10x =2, 10–20x =3, 20–30x=4, >30x=5). Hand cream application frequency: 0x =1, 1x=2, 1–2x=3, 2–5x=4, 5–10x=5, > 10x=6).

**TABLE 6:** Daily frequency of hand washing according to treatment of COVID‐19 patients

		Non‐COVID n (%)	COVID n (%)	Total n (%)	*P*‐value
Before COVID‐19 pandemic	<5x	14 (18.9)	8 (20.5)	22 (19.5)	
	5‐10x	34 (45.9)	14 (35.9)	48 (42.5)	
	10‐20x	22 (29.7)	12 (30.8)	34 (30.1)	
	20‐30x	4 (5.4)	4 (10.3)	8 (7.1)	
	>30x	0 (0)	1 (2.6)	1 (0.9)	
	Total	74 (100)	39 (100)	113 (100)	
	Mean ± SD	2.22 (.815)	2.38 (1.016)	2.27 (.889)	.45
During COVID‐19 pandemic	<5x	4 (5.5)	0 (0)	4 (3.5)	
	5‐10x	14 (19.2)	14 (3)	28 (24.8)	
	10‐20x	31 (42.)	13 (32.5)	44 (38.9)	
	20‐30x	21 (28.8%)	10 (25)	31 (27.4)	
	>30x	3 (4.1)	3 (7.5)	6 (5.3)	
	Total	73 (10)	40 (100%)	113 (100)	
	Mean ± SD	3.07 (.933)	3.05 (.959)	3.06 (.938)	.70

Abbreviation: SD, standard deviation.

*Note*: Mean: <5x =1, 5–10x =2, 10–20x =3, 20–30x=4, > 30x=5.

With regard to all HCWs, we found that the overall frequency of hand disinfection increased significantly after the COVID‐19 outbreak (before COVID‐19: median 10–20x per day, during COVID‐19: median 20–30x per day; *P* < .001) (Table 5). Detailed analysis of the Non‐C and C group showed slightly higher rates of disinfection in HCWs treating COVID‐19 patients, however, without significance (*P* = .09) (Table 7).

**TABLE 7:** Daily frequency of hand disinfection according to treatment of COVID‐19 patients

		Non‐COVID n (%)	COVID n (%)	Total n (%)	*P‐*value
Before COVID‐19 pandemic	<5x	5 (6.8)	1 (2.6)	6 (5.3)	
	5‐10x	20 (27)	7 (17.9)	27 (23.9)	
	10‐20x	24 (32.4)	17 (43.6)	41 (36.3)	
	20‐30x	20 (27)	4 (10.3)	24 (21.2)	
	>30x	5 (6.8)	10 (25.6)	15 (13.3)	
	Total	74 (100)	39 (100)	113 (100)	
	Mean ± SD	3.00 (1.05)	3.38 (1.14)	3.13 (1.09)	.13
During COVID‐19 pandemic	<5x	2 (2.)	0 ()	2 (1.8)	
	5‐10x	4 (5.4)	2 (5.1)	6 (5.)	
	10‐20x	24 (32.4)	6 (15.4)	30 (26.5)	
	20‐30x	23 (31.1)	17 (43.6)	40 (35.4)	
	>30x	21 (28.4)	14 (35.9)	35 (3)	
	Total	74 (100)	39 (100)	113 (100)	
	Mean ± SD	3.77 (1.01)	4.10 (0.85)	3.88 (0.971)	.09

Abbreviation: SD, standard deviation.

*Note*: Mean frequency of hand disinfection: <5x =1, 5–10x =2, 10–20x =3, 20–30x=4, > 30x=5.

### 3.5 Application of hand creams

When comparing the overall frequency of hand cream application before and during the COVID‐19 pandemic, our data indicate that application of skin care increased significantly from a mean of 1x per day to 1–2x daily (*P* < .001) (Table 5). HCWs in the Non‐C group applied hand cream more frequently compared to the C group (*P* = .074) (Table 8).

**TABLE 8:** Daily frequency of hand cream application according to treatment of COVID‐19 patients

		Non‐COVID n (%)	COVID n (%)	Total n (%)	*P*‐value
Before COVID‐19 pandemic	0x	15 (20.3)	9 (22.)	24 (21.1)	
	1x	18 (24.3)	10 (25%)	28 (24.)	
	1‐2x	22 (29.7)	13 (32.)	35 (30.7)	
	2‐5x	15 (20.3)	7 (17.5)	22 (19.3%)	
	5‐10x	3 (4.1)	1 (2.5)	4 (3.5)	
	>10x	1 (1.4)	0 (0)	1 (0.9)	
	Total	74 (100)	40 (100)	114 (100)	
	Mean (±SD)	1.68 (1.21)	1.53 (1.109)	1.62 (1.17)	.58
During COVID‐19 pandemic	0x	5 (6.8)	7 (17.5)	12 (10.5)	
	1x	9 (12.2)	6 (15)	15 (13.2)	
	1‐2x	16 (21.6)	10 (25)	26 (22.8)	
	2‐5x	25 (33.8)	9 (22.5)	34 (29.8)	
	5‐10x	15 (20.3)	7 (17.5)	22 (19.3)	
	>10x	4 (5.)	1 (2.5)	5 (4.4)	
	Total	74 (100)	40 (100)	114 (100)	
	Mean (±SD)	2.65 (1.28)	2.15 (1.42)	2.47 (1.34)	.074

Abbreviation: SD, standard deviation.

*Note*: Mean: 0x =1, 1x=2, 1–2x=3, 2–5x=4, 5–10x=5, > 10x=6).

## 4 DISCUSSION

During the ongoing COVID‐19 pandemic, first scientific reports have identified skin injuries in “front line” HCWs related to PPE use and intensified hand hygiene regulations.^16^, ^17^, ^18^, ^19^ However, the current situation is likely to cause an increase in cutaneous complications in all HCWs, regardless of direct treatment of COVID‐19 patients. Accordingly, increasing HCWs´ awareness of this disease is vital and the need for preventive measures cannot be over stressed.^20^, ^21^

The present study revealed a highly significant increase in hand washing and disinfection frequency across all HCWs after the start of the COVID‐19 outbreak, indicating that the proposed hygiene regulations have been implemented across all investigated disciplines (HCWs at a surgical clinic vs ICU). We found no significant difference with regard to the frequency of hand sanitation when comparing the Non‐C and C group, further stressing that the increase of hygienic safety precautions affects HCWs in general. In terms of hand disinfection, our data revealed slightly higher rates in the C group (*P* = .09), which could be related to the frequent change of PPE with hand disinfection involved in every step, (ie, when changing protective face visors, masks, gloves and gowns).

As hypothesized, our data show a high prevalence of characteristic symptoms for acute hand eczema^22^ across all HCWs, regardless of belonging to the Non‐C or C group (Non‐C: 93.2% vs. C: 85.0%, *P* = .19). For instance, dryness of hands was reported in >80% of HCWs and ~ 40% suffered from erythema. Symptoms characteristic of a chronic state such as fissures or pain were reported only in 9.6% and 4.4% of all cases, respectively. Thus, our findings add to the current data of Lan et al, who found an increasing onset of skin damage of the hands in 74.5% of 526 front‐line COVID‐19 HCWs, especially in those who washed their hands more than 10 times per day.^18^ Similarly, Yan et al reported that HCWs in China were vulnerable to skin and mucosal barrier breakdown due to frequent cleansing and long‐term use of PPE, while combating COVID‐19. Accordingly, they state that preventive hygiene measures caused acute and chronic dermatitis, secondary infections, and aggravation of existing skin diseases.^19^ Here, we show that the high frequency of hand hygiene also strongly affects hand skin status of workers who are not directly involved in the care of COVID‐19 patients.

Interestingly, only 15% of our study population stated they had hand eczema, although a majority reported symptoms associated with the disease. Similarly, Ibler et al previously found the notification rate of OSD to be as low as 12% in professions with clearly documented work‐related irritant exposures to the skin.^13^ Onset of hand eczema is likely to be associated with the intensified hand hygiene measures, as 83.3% of affected participants stated onset of the disease within the last 50 days. Studies like ours are thus indispensable to increasing awareness of this common, yet under‐recognized problem.

Currently, studies are focusing on proposing measures to prevent hand eczema during the pandemic. For instance, the use of sanitizers with ethanol as the main component is recommended, and application of moisturizers after hand washing.^19^ Cavanagh et al suggest abstention from overly hot water for handwashing and prolonged glove use and propose regular application of hypoallergenic moisturizers and hand sanitizers.^23^

In line with this, we found a higher frequency of hand cream application after COVID‐19 outbreak for all HCWs. However, although frequency of skin care increased from 1x to 1‐2x daily, rates are still rather low. Diepgen et al recommend the frequent application of lipid rich moisturizers during the working day and especially after work and at night.^22^ Based on evidence from clinical and experimental studies, skin protection in general should include protective gloves when performing wet work with a cotton glove worn underneath when glove use is expected to be longer than 10 minutes. Hands should be washed in lukewarm, not hot, water and dried thoroughly.^24^, ^25^ Interestingly, the Non‐C group showed significantly higher rates of hand cream application as compared to the C group. Time is a scarce resource in intensive care and accessibility might also be an issue, which could account for lower rates of skin care in the C group.

Skincare measures should be taken very seriously to prevent a potential chronic and relapsing state of hand eczema,^26^ potentially resulting in HCWs not being able to work. In this regard, surgeons and surgical nurses are at a special risk since their hands are central to their profession. Occlusive surgical gloves and frequent use of disinfectant detergents place these HCWs in a high risk group for onset of the disease.^12^ Furthermore, the damaged skin barrier and the pro‐inflammatory milieu present in irritant contact dermatitis^27^ can induce consecutive allergic contact sensitization,^28^ which is highly relevant for surgeons.^12^, ^29^, ^30^ OSDs are already amongst the most common work‐related diseases in industrialized countries, accounting for major health and economic expenses.^6^, ^31^, ^32^, ^33^ In the current situation, where we face a pandemic that will take months, if not years, to tackle, a loss of doctors and nurses would be devastating, especially, since preventive measures can be taken, and are easily applicable.

The sample investigated in this study reflects the overall distribution amongst HCWs with regard to gender (38.6% males, 61.4% females) and profession (34.2% physician, 65.8% nurses). The two groups (Non‐C and C) showed no significant differences regarding type IV hypersensitivity, atopic diathesis, or smoking status, and were therefore comparable. Interestingly, we observed a significant age difference between both groups. HCWs working in intensive care of COVID‐19 patients were significantly younger compared to the Non‐C group. This might point towards a preferred allocation of relatively younger staff to infectious ICU areas, to protect older personnel as a potential risk group.

To the best of our knowledge, this is the first study comparing the onset of hand eczema and associated symptoms during the COVID‐19 pandemic between HCWs directly involved in intensive care of COVID‐19 patients and HCWs without direct contact to this patient cohort in a single surgical clinic. This study is limited by the relatively small sample size. In the future, prospective studies should collect pooled data of large centers to further elucidate the onset of occupational hand eczema regarding different medical wards, working hours, shifts, and environmental factors. Furthermore, future studies should explore whether symptoms can be related to certain hand sanitizing products, in order to determine whether one product is preferable to another.

## CONFLICTS OF INTEREST

The authors declare no conflicts of interest.

## AUTHOR CONTRIBUTIONS

**Anne Guertler:** Conceptualization; data curation; formal analysis; investigation; methodology; visualization; writing‐original draft; writing‐review and editing. **Nicholas Moellhoff:** Conceptualization; data curation; formal analysis; investigation; methodology; visualization; writing‐original draft; writing‐review and editing. **Thilo Schenck:** Conceptualization; data curation; methodology; supervision; validation. **Christine Hagen:** Data curation; investigation. **Benjamin Kendziora:** Formal analysis; methodology. **Riccardo Giunta:** Conceptualization; supervision; validation; writing‐review and editing. **Lars French:** Conceptualization; supervision; validation; writing‐original draft; writing‐review and editing. **Markus Reinholz:** Conceptualization; methodology; supervision; validation; writing‐original draft; writing‐review and editing.
